# A Lightweight Pathological Gait Recognition Approach Based on a New Gait Template in Side-View and Improved Attention Mechanism

**DOI:** 10.3390/s24175574

**Published:** 2024-08-28

**Authors:** Congcong Li, Bin Wang, Yifan Li, Bo Liu

**Affiliations:** 1 College of Information Science and Technology, Hebei Agricultural University, Baoding 071001, China; boliu@hebau.edu.cn; 2 Hebei Key Laboratory of Agricultural Big Data, Baoding 071001, China; 20227060847@pgs0.hebau.edu.cn (B.W.); 20227060828@pgs0.hebau.edu.cn (Y.L.)

**Keywords:** abnormal gait recognition, transfer learning, classification of pathologies, ICBAM, MobileNetv2

## Abstract

As people age, abnormal gait recognition becomes a critical problem in the field of healthcare. Currently, some algorithms can classify gaits with different pathologies, but they cannot guarantee high accuracy while keeping the model lightweight. To address these issues, this paper proposes a lightweight network (NSVGT-ICBAM-FACN) based on the new side-view gait template (NSVGT), improved convolutional block attention module (ICBAM), and transfer learning that fuses convolutional features containing high-level information and attention features containing semantic information of interest to achieve robust pathological gait recognition. The NSVGT contains different levels of information such as gait shape, gait dynamics, and energy distribution at different parts of the body, which integrates and compensates for the strengths and limitations of each feature, making gait characterization more robust. The ICBAM employs parallel concatenation and depthwise separable convolution (DSC). The former strengthens the interaction between features. The latter improves the efficiency of processing gait information. In the classification head, we choose to employ DSC instead of global average pooling. This method preserves the spatial information and learns the weights of different locations, which solves the problem that the corner points and center points in the feature map have the same weight. The classification accuracies for this paper’s model on the self-constructed dataset and GAIT-IST dataset are 98.43% and 98.69%, which are 0.77% and 0.59% higher than that of the SOTA model, respectively. The experiments demonstrate that the method achieves good balance between lightweightness and performance.

## 1. Introduction

Neurological disorders are common in middle-aged and elderly people, and gait abnormalities are an important manifestation of neurological disorders. It has been noted that the prevalence of gait disorders increases from 10% in people aged 60–69 years to more than 60% in community residents aged 80 years and older [1]. Normal gait maintains stable limb mobility and complementary periodic movements under the control of the entire nervous system [2]. Abnormal gait is an involuntary behavior made by the body and controlled by neurological disorders, which can lead to weakened mobility and seriously affect the quality of life for patients. Patients with different neurological disorders have different gait patterns [3]. For example, patients with Parkinson’s disease often show festinating gait and freezing gait, hemiparetic patients have hemiparetic gait, and scissor gait is mostly seen in patients with spastic paraplegia. When diagnosing the above diseases, medical professionals often rely on qualitative evaluations such as personal professional experience [4], which makes it difficult to accurately assess the patients according to their actual conditions. Although the gait characteristics of walking in patients with the same disease are consistent with the characterization of the typical gait described above, the movements exhibited by each individual during walking can vary, making assessment relatively difficult. The development of telemedicine can avoid frequent hospital visits and further deterioration of patients’ conditions, but it is difficult for doctors to evaluate patients’ conditions by remote video. Gait recognition is an attractive biometric method designed to recognize people based on the way they walk [5]. Each person’s gait has unique characteristics such as stride length and limb movement, which make gait difficult to imitate. Therefore, the identification of patients’ gait through a high accuracy and lightweight abnormal gait recognition method is of great significance in assisting doctors in diagnosing the abovementioned neurological diseases and improving patients’ quality of life.

## 2. Related Work

Currently, abnormal gait recognition methods are mainly categorized into contact and noncontact. Contact abnormal gait recognition methods use more IMUs [6,7,8] and pressure sensors [9,10], which collect data to sense subtle changes in the lower limbs and soles. These methods have achieved better results in recognizing an abnormal gait, but the motion sensors are expensive, wearing and calibrating them requires professional guidance, and wearing the sensors affects the patient’s gait when walking.

The noncontact abnormal gait recognition method mainly uses various cameras to acquire images and then performs a series of processing on the images to obtain gait features, which eliminates the shortcomings of the contact method. In noncontact gait recognition methods, gait recognition methods based on skeletal data, gait recognition methods based on binary contour image sequences, and gait recognition methods based on energy images and other templates are more commonly used. With the advent of depth sensors (e.g., Kinect) and posture estimation methods, skeletal-based gait recognition has been used for the diagnosis, treatment, and rehabilitation of abnormal gait. Skeletal-based abnormal gait recognition methods are categorized into abnormal gait recognition based on the overall skeleton and based on joint point information. Tian et al. [11] proposed a method of abnormal gait recognition based on the overall skeleton and spatiotemporal attention-enhanced convolutional network of skeleton structure maps that can account for the spatial interactions of skeletal joints. Lee et al. [12] proposed an abnormal gait recognition method based on 3D joint point information for the multiKinects system and RNN-LSTM, which was able to recognize five types of abnormal gaits such as lurching gait, steppage gait, Trendelenburg gait, and so on. Although the above methods have achieved better results, the problem is that the skeletal data do not contain the shape and texture information of the human body, so the detailed information cannot be captured. Chen et al. [13] coded binary contour images into image vectors to distinguish between Parkinson’s patients and normal individuals. Albuquerque et al. [14] proposed a spatiotemporal deep learning method based on a sequence of contour images to recognize four abnormal gaits and one normal gait on the GAIT-IT dataset. Although the binary contour image contains the shape and texture information of the human body, the quality of its segmentation affects the classification results to a great extent. Methods based on templates such as energy images have more concise inputs and are easier to construct efficient algorithms than methods based on contour sequences [15]. In addition, the template-based approach effectively overcomes the limitation that the quality of binary contours affects the classification results. Common gait templates are the gait energy image (GEI) [16], gait entropy image (GEnI), active energy image (AEI), etc. Elkholy et al. [17] learned representative features from the GEI by convolutional self-encoder and used anomaly detection methods to detect an abnormal gait caused by diseases such as Parkinson’s disease and stroke, enabling multiangle gait disorder detection. Zhou et al. [18] proposed a novel method for estimating the physical impairment of elderly people using gait, which was realized by using a specific patch-GEI for the detection of visually impaired gait and leg-impaired gait. Albuquerque et al. [19] proposed a telemedicine healthcare system using a shallow CNN structure for automatic assessment of gait affected by pathologies such as diplegic, hemiplegic, Parkinson’s disease, and neuropathic. The GEI is superimposed on a cycle of normalized contour images and is calculated by averaging the images, which loses important temporal information. Bashir et al. [20] first proposed a new gait representation, the GEnI, which can capture most of the motion information and is robust to changes in various covariate conditions affecting appearance. The GEnI provides richer and more robust gait information than the GEI and AEI. The GEnI contains more spatial and temporal features about the gait, which can better capture the details of the gait, thus improving the robustness of gait recognition. Zhang et al. [21] first proposed a new gait representation method by accumulating the active region extracted from the difference between two neighboring silhouette images—AEI. The AEI focuses primarily on dynamic information in gait, but ignores static information, and contains very little profile information. In research on the template-based recognition of abnormal gait, there is little direct use of the GEnI or AEI to recognize abnormal gait.

For this reason, given that the strengths and weaknesses of the three images, GEI, GEnI, and AEI, can complement each other, in this paper, we choose to use batch gradient descent (BGD) to optimize the weights for the fusion of the three images in the side-view and generate the fused energy image (FEI) by fusing them according to the optimal fusion weights and using it as an input to our algorithm. Verlekar et al. [22] used a transfer learning approach to address the problems of overfitting and low accuracy of the VGG19 model on a small pathological gait dataset. Traditional deep learning gait recognition algorithms such as VGG19 mentioned above have large parameter counts and high computational complexity, which are significant drawbacks for resource-constrained devices. Therefore, for relatively small pathological gait datasets, the use of lightweight network models is a good solution. Based on a transfer learning method, we propose a lightweight pathological gait recognition network (NSVGT-ICBAM-FACN) that fuses attentional features and convolutional features. In this paper’s pathological gait recognition method, we improved the convolutional block attention module (CBAM) to obtain the module (ICBAM), which allows the network to focus on the important features and suppress unnecessary features. Compared with the use of squeeze-and-excitation (SE) [23], efficient channel attention (ECA) [24], and the CBAM, the ICBAM ensures the effectiveness of the module without adding much computational cost.

Currently, most abnormal gait recognition studies use sensor datasets of abnormal gait and image datasets of healthy subjects simulating abnormal gait (such as the INIT dataset [25], the DAI dataset [26], the DAI2 dataset [27], and the GAIT-IST dataset [28]). The INIT dataset simulated seven different gait disorders, but these gait disorders could not be directly deduced from the associated pathologies. The DAI and DAI2 datasets simulated abnormal gaits for four pathologies, but the number of abnormal gait sequences collected was very small. The GAIT-IST dataset provides Parkinsonian gait but does not subdivide it. So, we subdivided it into festinating gait and shuffling gait in freezing gait, which can identify Parkinson’s disease more finely. Therefore, we constructed an abnormal gait dataset. The main contributions are summarized below:

- By analyzing the advantages and disadvantages of the GEI, GEnI, and AEI, along with the relationship between their generation principles and abnormal gait characteristics, we design a comprehensive gait template that is robust and contains rich gait information.
- We propose an ICBAM that employs depthwise separable convolution (DSC) to replace ordinary convolution in the spatial attention module and connect it in parallel for capturing key features and richer discriminative information.
- We constructed an abnormal gait dataset that can recognize not only festinating gait and shuffling gait in Parkinson’s disease, but also pathological gait in hemiplegic disease and spastic paraplegic disease.
- A lightweight deep learning model, NSVGT-ICBAM-FACN, is used to evaluate pathological gait. The accuracy of this paper’s method is proven to be superior to other methods by ablation studies, module comparison experiments, and identification results on the self-constructed abnormal gait dataset and GAIT-IST dataset, which ensures high accuracy while keeping the model lightweight.

The paper is organized as follows: Section 3 introduces the gait cycle division module, the new cross-view gait template, and the details of the proposed model. Section 4 describes the dataset construction, evaluation indicators, settings, and the results of the experiment. Finally, we conclude in Section 5.

## 3. Materials and Methods

### 3.1. Overall Architecture of the Proposed Algorithm

**Figure 1:**
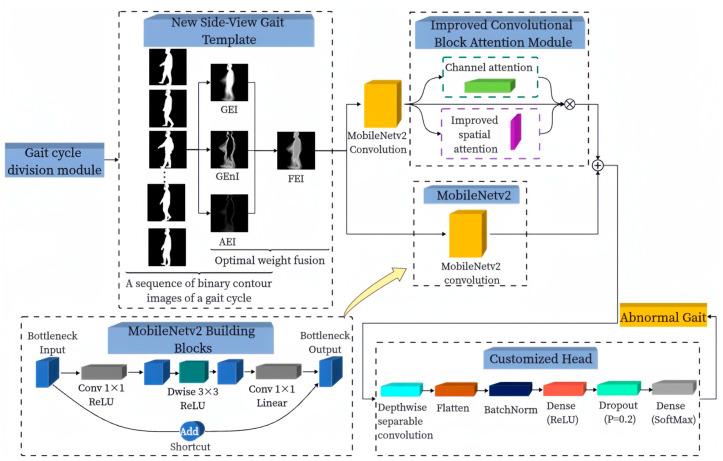
The overall architecture of the proposed algorithm.

Figure 1 illustrates the overall structure of the proposed algorithm for abnormal gait recognition outlined in this paper. We devised a gait cycle division module tasked with automatic gait cycle segmentation. From the resulting binary contour image sequences, we compute the GEI, GEnI, and AEI correspondingly. Optimizing the fusion weights of these three images via BGD allows us to attain the most optimal fusion weights. Subsequently, these optimized weights are applied to fuse the three images, culminating in the creation of the FEI. The FEIs are fed into the attention branch composed of the ICBAM and MobileNetv2 convolution module and the convolution branch of the MobileNetv2 convolution module, respectively, and the obtained attention features and convolution features are fused to obtain the fusion features. A new customized head is designed using operations such as global average pooling with DSC replacement, and the fused features are fed into this head for pathological gait recognition.

### 3.2. Gait Cycle Division Module

Human walking is a cyclic behavior, and the gait cycle is generally defined as the time elapsed from the time the foot hits the ground to the time the heel of the leg on the same side hits the ground again. Since the height of the person does not change when the person walks, and the width of the person changes periodically with the swinging of the legs, the width-to-height ratio of the person is utilized as a feature for periodic detection. Since the manual process of dividing the gait cycle is slow and cumbersome, we designed this module to divide the gait cycle automatically and use it to make the process of dividing the gait cycle more efficient.

**Figure 2:**
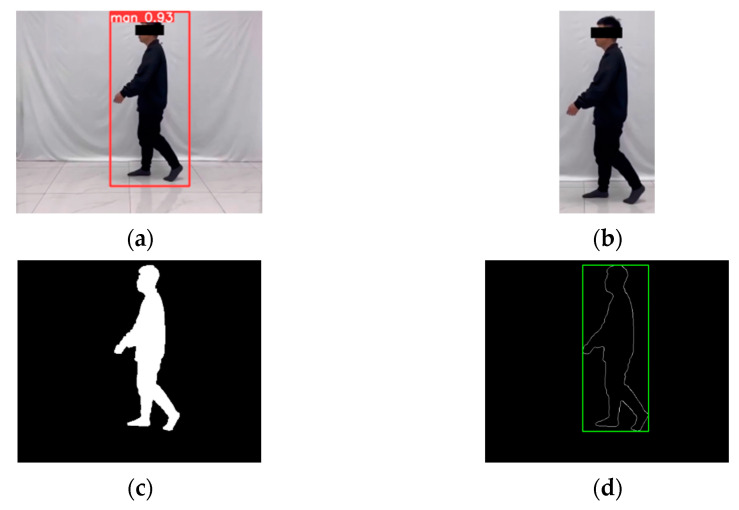
(**a**) Image with detection frame; (**b**) cropped image; (**c**) binary contour image; (**d**) human silhouette with the minimum external rectangle.

**Figure 3:**
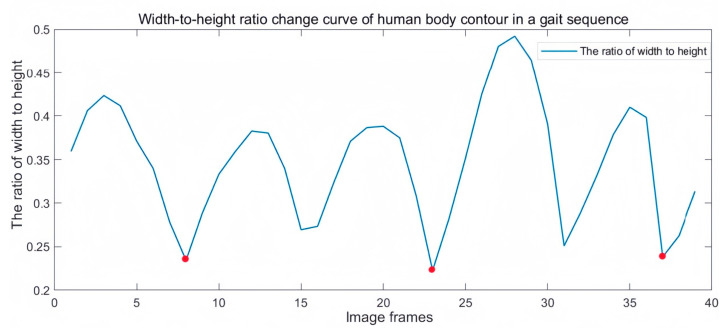
Width-to-height ratio change curve of a human body contour in a gait sequence.

Although the gait template designed in this paper can effectively manage the problem that the low quality of contour segmentation affects the classification results, to achieve more accurate recognition we also need to ensure the quality of contour segmentation. First, to avoid external factors such as background, illumination, and reflected light from the floor, which affect the segmentation quality of the silhouette, we use the YOLOv5 [29] target detection algorithm to automatically crop the human body regions detected in the image to facilitate finer contour segmentation. Then, the cropped image sequence is grayed out, binarized, and morphologically processed to obtain a binary contour image sequence. The binary contour image sequence is size-normalized to obtain a binary image sequence of 256 × 256 pixels. Finally, the contour of the human body is extracted by the Sobel edge detection method, and the gait cycle is detected and divided by calculating the width-to-height ratio of the smallest outer rectangle of the human body contour. The two adjacent troughs are half a gait cycle. It is verified that the gait cycle divided by this gait cycle division module matches the actual gait cycle. The images involved in this process are shown in Figure 2. The width-to-height ratio change curve of a human body contour in a gait sequence is shown in Figure 3.

### 3.3. New Gait Template

#### 3.3.1. Gait Energy Image (GEI)

The GEI was generated by averaging a sequence of binary contour images acquired through gait cycle segmentation. The GEI contains not only dynamic information about the human body as it walks, but also static information. During walking, areas with frequent movements have high energy and large pixel values. Conversely, the energy is low and the pixel values are small. The GEI is a global feature representation that captures information about the entire gait cycle. Therefore, the GEI is suitable for gait recognition of the overall process of the five gaits described in this paper. The GEI is calculated as follows:

(1) $$GEI\left(x,y\right)=\frac{1}{N}\sum_{t=1}^{N}F_{t}\left(x,y\right)$$

where N denotes the total number of frames in the gait image sequence, $F_{t}\left(x,y\right)$ denotes the binary contour image at moment t, and $GEI\left(x,y\right)$ denotes the GEI.

#### 3.3.2. Gait Entropy Image (GEnI)

Before generating the GEnI, we need to calculate the Shannon entropy for each pixel point location in the contour image sequence, which is calculated by the following formula:

(2) $$H\left(x,y\right)=-\sum_{j=1}^{K}p_{j}(x,y)\times \log_{2}p_{j}(x,y)$$

where, since the contour image we are using is a binary image, the value of K is taken as 2; x,y denotes the coordinates of the pixel point in the contour image, $p_{j}(x,y)$ denotes the probability of the pixel point to take the value of jth, and $H\left(x,y\right)$ denotes the Shannon entropy of the pixel point.

After calculating the Shannon entropy for each pixel point location in the sequence, a GEnI is generated. In the GEnI, the dynamic regions of the limbs have higher entropy values and are more informative, while the static portion of the torso has an entropy value of zero. The GEnI provides a wider range of feature representations, including gait complexity and variety. This makes it more suitable for detecting different types (variable speed, variable stride) of abnormal gait, such as festinating gait and shuffling gait. The GEnI is calculated as follows:

(3) $$GEnI\left(x,y\right)=\frac{(H\left(x,y\right)-H_{min})\times 255}{H_{max}-H_{min}}$$

where $H_{max}$ denotes the maximum value of Shannon entropy, $H_{min}$ denotes the minimum value of Shannon entropy, and $GEnI\left(x,y\right)$ denotes the GEnI.

#### 3.3.3. Active Energy Image (AEI)

In the adjacent anterior and posterior frames of people walking, the torso is nearly stationary, and limb movement is most prominent. So, the AEI discards the stationary part and extracts only the moving part. The higher the frequency of motion in the dynamic part, the stronger the pixel intensity in that part of the AEI. We can extract the dynamic part of the human body by calculating the difference between two neighboring frames of binary contour images in the same gait cycle. The AEI mainly captures the action features in the gait, such as step frequency, step length, and gait phase. Abnormal gait usually exhibits a different dynamic pattern than normal gait, so the dynamic features of the AEI are more appropriate for detecting festinating gait, scissor gait, and hemiplegic gait. The AEI is calculated as follows:

(4) $$AEI\left(x,y\right)=\frac{1}{N}\sum_{t=1}^{N}|F_{t}\left(x,y\right)-F_{t-1}\left(x,y\right)|$$

where $\left|F_{t}\left(x,y\right)-F_{t-1}\left(x,y\right)\right|$ denotes the extracted dynamic region and AEI(x,y) denotes the AEI.

#### 3.3.4. Batch Gradient Descent (BGD)

BGD [30] is a first-order optimization algorithm whose main objective is to find the minimum value of the objective function through iterations. In this study, the objective function we require is the cost function, which represents the degree of error between the target image and the estimated image. The cost change is the difference between the cost function of two neighboring iterations. If the cost change is less than a set threshold, or if the maximum number of iterations is reached, the iteration is stopped. The BGD is run multiple times using several different initial parameter values, and then the set of parameters with the smallest cost function value is selected as the final result. The GEI, GEnI, and AEI corresponding to different gait cycles have different optimal fusion weights. The formula for calculating the optimal fusion weights of the image is as follows:

(5) $$weights=\frac{1}{n_{images}}$$

(6) estimated_image=image_matrix×weights

(7) error=target_pixels−estimated_image

(8) $$cost=\sum_{i=1}^{n_{pixels}}{error}_{i}^{2}$$

(9) $$grad={image\_matrix}^{T}\times error$$

(10) $$weights=\frac{weights-learning\_rate\times grad}{\sum_{i=1}^{n_{images}}{weights}_{i}}$$

where image_matrix is a matrix that stores the GEI, GEnI, and AEI pixels; estimated_image denotes the current estimated weight matrix, resulting in an “approximate” reconstruction of the image by multiplying and summing the pixels; target_pixels denotes the pixel values of the target image and is used to store the sum of all the pixel values; error denotes the error between the target_pixels and the estimated_image; and cost denotes the degree of error between the target_pixels and the estimated_image. The ${image\_matrix}^{T}$ is the transpose of the image matrix, grad is the gradient, and weights is the current weight of each image.

#### 3.3.5. Fusion Energy Image (FEI)

To obtain an FEI with a size of 256 × 256 pixels, the GEI, GEnI, and AEI are fused according to the optimal weights obtained by the BGD.

### 3.4. NSVGT-ICBAM-FACN

Among the popular lightweight CNN algorithms, we choose MobileNetv2 as the basic algorithm due to its better balance between model speed and performance. Based on MobileNetV2, this paper proposes a lightweight deep neural network model NSVGT-ICBAM-FACN for recognizing multiple pathological gaits. In this model, the preprocessed gray-scale FEIs are input to both the single branch of the MobileNetv2 convolutional network and the combined branch of the MobileNetv2 convolutional network with the ICBAM. In a single branch, the MobileNetv2 convolutional network directly extracts the convolutional feature map. In the binding branch, the ICBAM focuses on the feature maps extracted by the MobileNetv2 convolutional network to generate the attention feature maps to obtain important information. The ICBAM helps to reduce the weight of irrelevant information in the image and reduces redundant information. The attention feature map and the convolutional feature map are then fused to obtain the fused feature map. 

Since the classification layer of the MobileNetV2 model obtained by training on the ImageNet dataset has 1000 nodes, a new customized head with 5 classifications was designed to match the experimental content in this study. The customized head contains (i) DSC, (ii) flatten layer, (iii) dense layer 1, (iv) BN layer, (v) ReLU activation function, (vi) dropout layer, (vii) dense layer 2, and (viii) SoftMax layer. In the last feature map of the original 7 × 7 global average pooling layer, the perceptual domain of the center point and the perceptual domain of the edge points are the same, but the perceptual domain of the center point includes the complete picture, while the perceptual domain of the edge points is only a part of the picture, so the weight of each point should be different, but the average pooling layer takes them into account as if they were the same, and therefore the network performance will decrease. We chose to replace the global average pooling layer in the original model with a DSC consisting of a depthwise convolution with a convolutional kernel size of 3 × 3 and a pointwise convolution so that the network can learn the weights at different points. On the one hand, the number of output channels is reduced and the quality of the feature representation per channel is higher. On the other hand, the spatial information is maintained, and there is also some regularization effect, which enhances the feature representation and the generalization capability of the model. Adding a BN layer to batch-normalize the output of the previous layer helps to increase the training speed of the model and enhance its robustness. Adding the ReLU activation function behind the BN layer introduces nonlinearities that allow the model to learn more complex features. We chose to include a dropout layer before the final fully connected layer to randomly deactivate neurons during training and to further prevent model overfitting. The combination of these operations allows the model to improve its performance and stability without disproportionately increasing the number of parameters.

### 3.5. MobileNetv2

**Figure 4:**
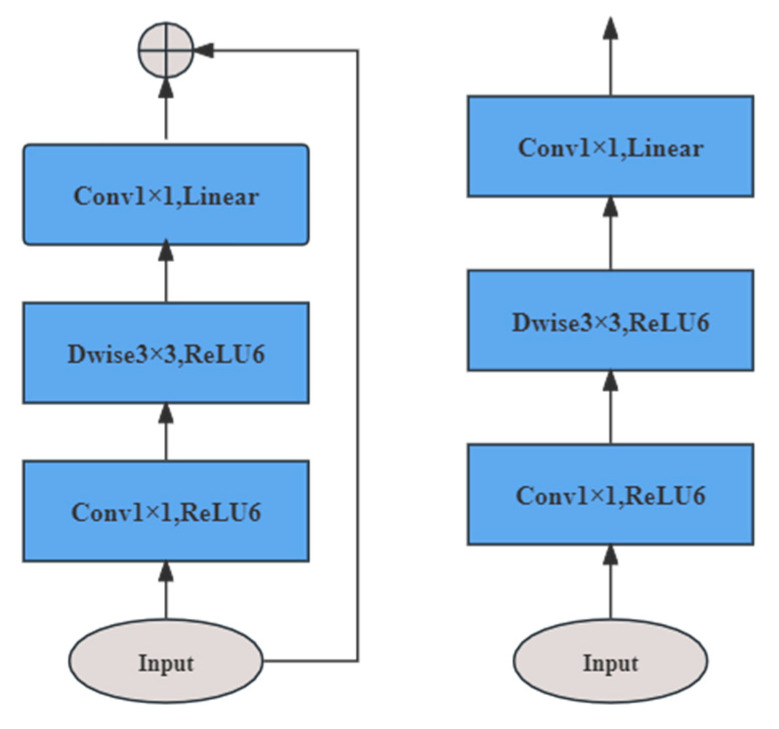
Structure of the inverted residual block.

MobileNetV2 [31] is a lightweight convolutional neural network for mobile devices developed by a Google team in 2018. MobileNetV2 exhibits a smaller model size, less computation, and higher computational efficiency than conventional convolutional neural networks. As shown in Figure 4, the main characteristics of MobileNetV2 are as follows: (i) Inverted residual structure. First, pointwise convolution is used for dimension upgrading, then 3 × 3 depthwise convolution feature extraction, and finally pointwise convolution for dimension downgrading, which improves the feature expressiveness and the depth of the model while reducing the amount of computation and the number of parameters. Reduced storage requirements make the model more efficient on resource-constrained devices. (ii) Linear bottleneck structure. Since the inverted residual structure outputs low-dimensional feature information, a linear activation function is used to avoid feature loss. (iii) DSC. The DSC reduces the number of parameters and computation in the model while achieving the same effect as the standard convolution for feature extraction. (iv) Shortcut connection. This design helps to spread the gradients and facilitates the transfer and reuse of features.

### 3.6. Improved Convolutional Block Attention Module

**Figure 5:**
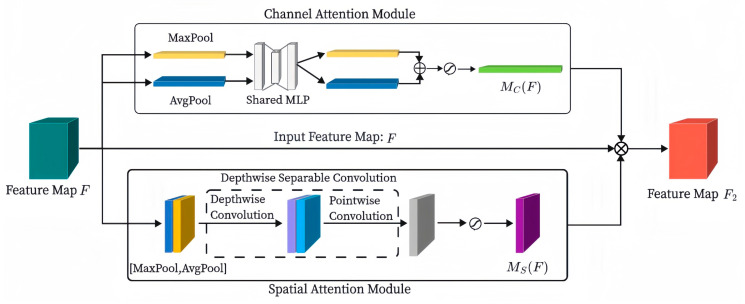
Improved convolutional block attention module.

Woo et al. [32] proposed a lightweight CBAM, which can be seamlessly integrated into any CNN architecture, can be trained end-to-end with the underlying CNN, and has negligible overhead. The CBAM contains the channel attention module and the spatial attention module, and since the two submodules work through a serial connection, the inputs to the features in the spatial attention module, which is in the back of the queue, are affected to some extent by the channel attention module in front of it. Therefore, the overall structure of the CBAM is modified to parallel connection, so that the two attention modules can directly learn the original feature inputs without being affected by the order of the front and back of the channel attention and spatial attention modules. Since depthwise convolution learns the spatial features of the input data, while pointwise convolution learns the relationships between channels, DSC can better capture complex features. The relatively small number of output channels of the DSC means the computation of convolutional operations in the inference phase is faster, which is useful for applications with high real-time requirements (e.g., real-time image processing). Therefore, we replace the ordinary convolution in the spatial attention module with a convolution kernel size of 7 × 7 with a DSC consisting of a depthwise convolution with a convolution kernel size of 7 × 7 and a pointwise convolution, and then we obtain an ICBAM. The ICBAM has improved performance with almost no increase in the number of parameters and is better than the CBAM. The detailed structure of the ICBAM is shown in Figure 5.

The formula for the channel attention module is as follows:

(11) $$M_{c}\left(F\right)=\sigma \left(MLP\left(Avgpool\left(F\right)\right)+MLP\left(Maxpool\left(F\right)\right)\right)=\sigma \left(W_{1}\left(W_{0}\left(F_{avg}^{c}\right)\right)+W_{1}\left(W_{0}\left(F_{max}^{c}\right)\right)\right)$$

where σ denotes the Sigmoid nonlinear activation function, MLP denotes the fully connected hidden layer and fully connected output layer, and $W_{1}$ and $W_{0}$ represent the weight matrices. $F_{avg}^{c}$ and $F_{max}^{c}$ denote the average pooling feature and the maximum pooling feature on the channel dimension, respectively. 

The formula for the spatial attention module is as follows:

(12) $$M_{s}\left(F\right)=\sigma \left(f_{p}^{1\times 1}\left(f_{d}^{7\times 7}\left(\left[Avgpool\left(F\right);\;Maxpool\left(F\right)\right]\right)\right)\right)=\sigma \left(f_{p}^{1\times 1}\left(f_{d}^{7\times 7}([F_{avg}^{s};F_{max}^{s}])\right)\right)$$

where $f_{d}^{7\times 7}$ denotes a depthwise convolution operation with a convolution kernel size of 7 × 7 and $f_{p}^{1\times 1}$ denotes the pointwise convolution operation. $F_{avg}^{s}$ and $F_{max}^{s}$ denote the average pooling feature and the maximum pooling feature on the spatial dimension, respectively.

The formula for the ICBAM is as follows:

(13) $$F_{2}=M_{C}(F)⊗M_{S}(F)⊗F$$

where ⊗ denotes the elementwise multiplication, F denotes the input feature map, and $F_{2}$ denotes the final feature map generated by F after the ICBAM. $M_{c}\left(F\right)$ and $M_{s}\left(F\right)$ denote channel attention features and spatial attention features, respectively.

### 3.7. Transfer Learning

Transfer learning [33] is a machine learning method that applies the weights of a convolutional neural network model trained on one domain or task to another new domain or task. To avoid the model learning from scratch, to help the model better understand the characteristics of the current task, and to improve the model performance, this study adopts the MobileNetv2 model, which has been trained on the large-scale dataset ImageNet [34]. As the base model, the classifier weights were removed, feature weights frozen, and newly added layers retrained.

## 4. Experiment and Result Analysis

### 4.1. Dataset Construction

#### 4.1.1. Characterization of the Five Gait Types

Patients with different neurological disorders have different gait characteristics, and physicians often use different gait characteristics to assess and determine the patient’s disease. A common gait in people with Parkinson’s disease is the festinating gait and the frozen gait. Frozen gait is divided into three main categories: (1) shuffling gait; (2) tremor-in-place type; and (3) complete immobility. In contrast to tremors in place and complete immobility, people with shuffling symptoms have a milder condition, can usually walk without assistance, and timely detection and treatment can prevent the condition from worsening. Compared with tremor in place and total immobility, in patients with shuffling symptoms, the condition is less severe and they are usually able to walk without the help of others, and timely detection and treatment can prevent the condition from worsening. Therefore, among the frozen gait, the shuffling gait is selected for study in this paper. Eventually, we chose to use festinating gait, scissor gait, hemiparetic gait, shuffling gait, and normal gait as the subjects of study to identify Parkinson’s disease, hemiplegia disease, spastic paraplegia disease, and healthy subjects. Table 1 shows the different walking characteristics for different gaits.

#### 4.1.2. Experimental Scheme

**Figure 6:**
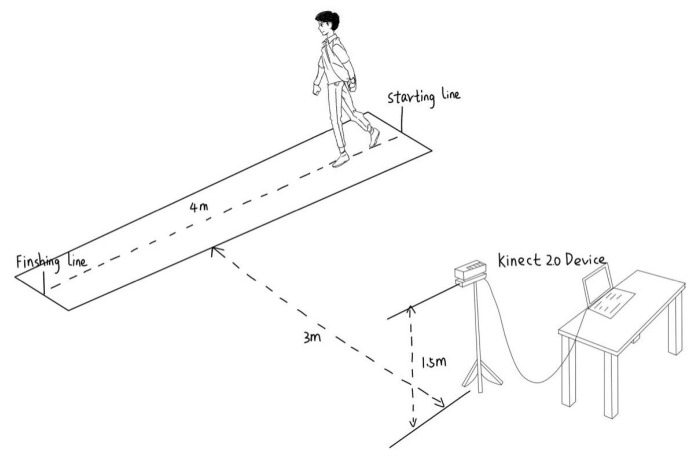
Experimental environment.

**Figure 7:**
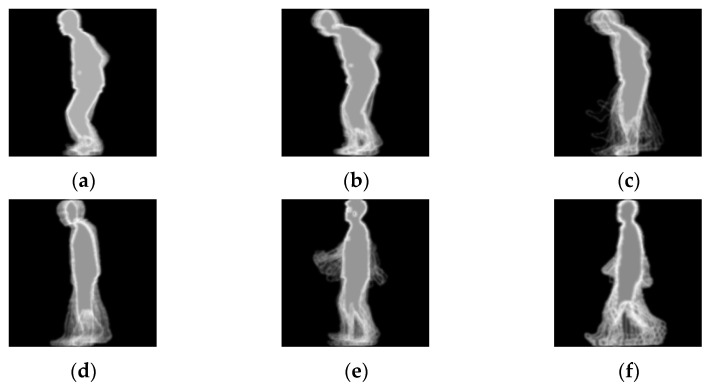
Sample image. The first row is in order from left to right: (**a**) festinating gait; (**b**) scissor gait; and (**c**) hemiparetic gait. The second row is in order from left to right: (**d**) shuffling gait; (**e**) normal gait; and (**f**) normal gait in the CASIA-B dataset.

Informed consent was obtained from all subjects involved in the study. The experimental environment is shown in Figure 6. In this paper, color image sequences of the side-view gait were captured using a Kinect 2.0 device with 1080P resolution and stable performance, and the device was mounted on a 1.5 m high tripod. Subjects walked within a distance of 4 m and remained 3 m from the device. One normal gait and four abnormal gaits simulating festinating gait, scissor gait, paraplegic gait, and shuffling gait were collected for this paper. In this paper, we refer to the characterization of the five gait types [35,36,37] and instruct 18 subjects (aged 20–25 years) to simulate the four abnormal gaits. Each individual walked from right to left six times, with at least six gait sequences of each gait for each individual. We chose the FEI generated for each gait cycle as a sample. Since there are fewer samples of normal gait with the same gait sequence, and the various types of samples are not well balanced, we refer to the CASIA gait dataset to extend the dataset [38]. The gait sequences of nine individuals were selected in subdataset B of the CASIA gait dataset and 180 normal gait samples were generated. In this dataset, the sample sizes for festinating gait, scissor gait, hemiparetic gait, shuffling gait, and normal gait were 264, 192, 236, 254, and 341, respectively. The sample size was 256 × 256 pixels. Figure 7 shows the corresponding examples for each gait and normal gait in the CASIA-B dataset.

### 4.2. Evaluation Indicators

To further validate the effectiveness of the method described, this study uses accuracy (Acc), precision (Prec), sensitivity (Sens), specificity (Spec), and macro F1-score (MF1) as the evaluation indexes, and the index formulas are as follows:

(14) $$Accuracy=\frac{1}{N}\sum_{i=1}^{n}{TP}_{i}$$

(15) $$Precision=\frac{1}{n}\sum_{i=1}^{n}\frac{{TP}_{i}}{{TP}_{i}+{FP}_{i}}$$

(16) $$Sensitivity=\frac{1}{n}\sum_{i=1}^{n}\frac{{TP}_{i}}{{TP}_{i}+{FN}_{i}}$$

(17) $$Specificity=\frac{1}{n}\sum_{i=1}^{n}\frac{{TN}_{i}}{{TN}_{i}+{FP}_{i}}$$

(18) $$MF1=\frac{1}{n}\sum_{i=1}^{n}\frac{2\;*{Precision}_{i}\;*\;{Recall}_{i}}{{Precision}_{i\;}+{Recall}_{i}}$$

where TP, TN, FP, and FN represent true positive, true negative, false positive, and false negative. N is the total number of samples, I is the i-th class, and n is the number of gait classes.

To select the optimal module and model, we also evaluated the number of parameters, number of floating point operations per second (Flops), memory usage, and frames per second (FPS) for the different modules and models.

### 4.3. Settings

This experiment is based on the Windows 11 operating system, the GPU is NVIDIA GeForce RTX 3050, the processor is AMD Ryzen 7 5800H with Radeon Graphics 3.20 GHz, and the running memory is 16 GB. The Python version is 3.7.1, and the deep learning framework is Pytorch. The parallel computing framework and version is CUDA version 11.4, and the development environment is Pycharm.

The dataset is randomly divided proportionally into training and testing sets, with 80% of its data used for model training and 20% for model testing. Adam is chosen for the model optimizer and cross-entropy is used for the loss function. The learning rate was set to 0.0001, the batch size to 32, and the number of training rounds to 100. To prevent overfitting, the dropout value was set to 0.2.

### 4.4. Ablation Study

In this study, ablation experiments are designed to demonstrate that each of the improvements made in MobileNetv2 contributes to improving the performance of the model. This ablation experiment focuses on the adoption of dual branching, the incorporation of the ICBAM, the use of DSC in place of global average pooling, and the design of customized heads. Table 2 shows the design of the five sets of experiments (numbered 1–5).

**Figure 8:**
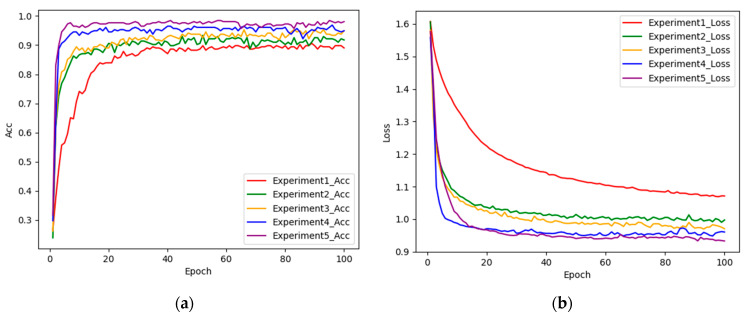
(**a**) Curve of change in accuracy for each set of experiments; (**b**) curve of change in loss value for each set of experiments.

Table 3 displays the results obtained from each set of experiments. In Experiment 2, the incorporation of the ICBAM following the convolutional layer in the original MobileNetv2 model from Experiment 1 resulted in a 2.74% accuracy enhancement. This signifies that the ICBAM enables dynamic focus on various regions within the input feature map, thereby aiding in the comprehensive capture of pertinent semantic information. Experiment 3 involved the transformation of a single branch into a dual branch, leading to overall metric improvement. This underscores that the fusion of attention features and convolutional features amplifies feature representation, thereby fortifying the model’s robustness and generalization capacity. Experiment 4 substituted global average pooling with DSC, demonstrating improved spatial information capture and interchannel correlation, facilitating enhanced feature learning while retaining spatial details. Experiment 5 introduced a novel customized header, expediting training convergence, diminishing gradient issues, and mitigating overfitting concerns. These structural modifications amalgamate to form the model proposed in this study, elevating recognition accuracy on the gait dataset by 7.84% compared to the original network model, showcasing enhanced stability and performance. The final model exhibits superior values across all metrics, highlighting its robustness, stability, and exceptional overall performance. Figure 8 illustrates the change curves of accuracy and loss values following each model enhancement, indicating an enhanced convergence rate with each incremental component addition. Compared to the original MobileNetv2 model, the proposed algorithm herein manifests lower loss values and improved model convergence.

### 4.5. Comparative Performance Experiments with Attention Modules

To verify the performance of different attention modules, five-class classification experiments were performed on our dataset (image size: 256 × 256 pixels) and GAIT-IST (image size: 224 × 224 pixels), respectively, and the experimental results are shown in Table 4. The experimental results show that (i) in our dataset, our attention module improves the accuracy over ECA, SE, and the CBAM by 1.57%, 1.18%, and 0.78% respectively. (ii) For GAIT-IST, the accuracy displays increments of 0.64%, 0.32%, and 0.32% over ECA, SE, and the CBAM, correspondingly, where SE and the CBAM have the same accuracy. In terms of the number of parameters, the number of parameters in our attention module increases by 0.21 M compared to ECA, which is comparable to the number of SE and CBAM parameters. From the results, it can be concluded that ECA focuses on attention in the channel dimension, while SE and the CBAM focus on joint attention in the spatial and channel dimensions. They do not have explicit parallel convolutional branches and DSC, and may not be able to capture more complex features. Our attention module has an increased number of parameters compared to ECA, but remains lightweight and has higher accuracy.

### 4.6. Comparative Experiments on the Validity of Different Components in the New Gait Template

We designed comparison experiments of different components in the new gait template to demonstrate the effectiveness of the new gait template. Each component was recognized by the NSVGT-ICBAM-FACN network and the results are shown in Table 5. From the table, we can see that the accuracy of recognition by the GEnI is higher than that for the AEI and GEI, which indicates that the GEnI provides richer information and is more robust. The recognition effect of the FEI is better than that of the three images individually, which indicates that the shortcomings of each of the three images are complementary after fusion, and it proves feasibility that the three images are fused according to the optimal fusion weights optimized by the BGD.

### 4.7. Compared with the State-of-the-Art Models

As shown in Table 6, our method is compared with other state-of-the-art methods on GAIT-IST and our datasets, respectively. (i) GAIT-IST dataset. Using the skeleton energy image (SEI), the increase in the number of parameters and the number of Flops of this paper’s model is insignificant compared to GhostNet, with an increase of 0.29 and 0.146, respectively, with comparable accuracy. In comparison to the fine-tuned VGG-19, our proposed model exhibits a substantial reduction in parameters by roughly 48 times, Flops by 61 times, and memory usage by 47.5 times. Additionally, it boasts a 34.87 increase in FPS and a 0.64% enhancement in accuracy. Notably, the accuracy of using the FEI in this paper is higher compared to the SEI, with an improvement of 0.65%, indicating that the FEI contains richer discriminative information. (ii) Our dataset. When using the FEI, compared to the fine-tuned VGG-19, our proposed model exhibits a substantial reduction in parameters by roughly 46 times, Flops by 46.7 times, and memory usage by 55.7 times. Additionally, it boasts a 43.95 increase in FPS and a 0.77% enhancement in accuracy. From the above results, it can be concluded that our method performs well on both the GAIT-IST dataset and our dataset, and achieves a good balance between lightweightness and performance.

### 4.8. Experimental Results of the Model on the Self-Constructed Dataset

**Figure 9:**
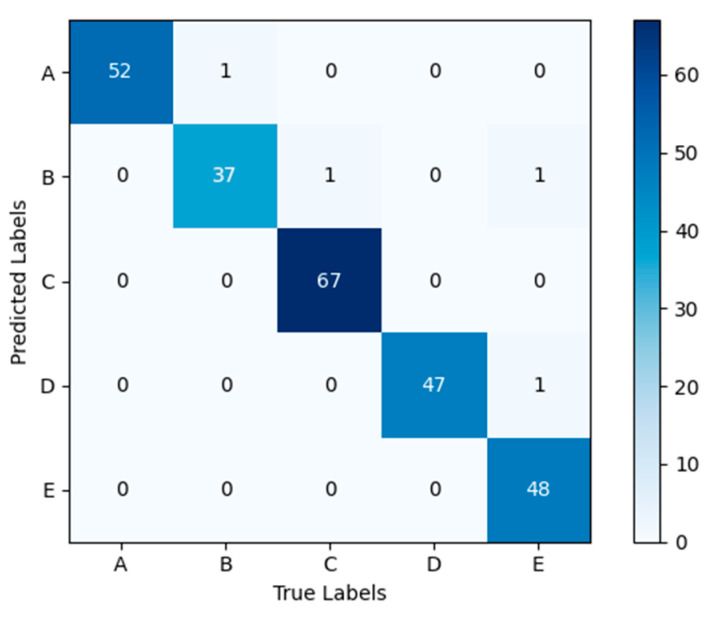
Confusion matrix of the model on the self-constructed dataset. (A: festinating gait; B: scissor gait; C: normal gait; D: hemiparetic gait; E: shuffling gait).

The confusion matrix obtained by applying the model NSVGT-ICBAM-FACN to the self-constructed gait dataset used in this study is shown in Figure 9. The confusion matrix shows the number of samples correctly and incorrectly predicted for each of the five gaits in the test set. The values on the main diagonal of the confusion matrix indicate the number of samples correctly categorized by the model during the prediction process, i.e., the true category is the same as the predicted category. Values in other positions indicate the number of samples misclassified by the model. The confusion matrix shows that a normal gait and a shuffling gait were incorrectly recognized as scissor gait. This may be because the magnitude of the movement while walking is too small, causing the model to make an error in the recognition.

Usually, models perform well with trained data and poorly with real data. Therefore, we use untrained test-set images to test the performance of the model. Table 7 gives the test results of the NSVGT-ICBAM-FACN model in terms of precision, sensitivity, and specificity. The table shows that the model recognizes the gaits in the test set very well. The five gaits exhibit precision higher than 94.87%, sensitivity higher than 96%, and specificity higher than 99.08%, which indicates that the model described in this paper has a significant recognition effect on individual gaits and distinguishes the five gaits well. Taking shuffling gait as an example, the recognition precision and specificity reached 100%, indicating that the model performed ideally in the recognition task of shuffling gait, and was able to carry out the recognition perfectly without any error or omission.

### 4.9. Experimental Results of the Model on the GAIT-IST Dataset

**Figure 10:**
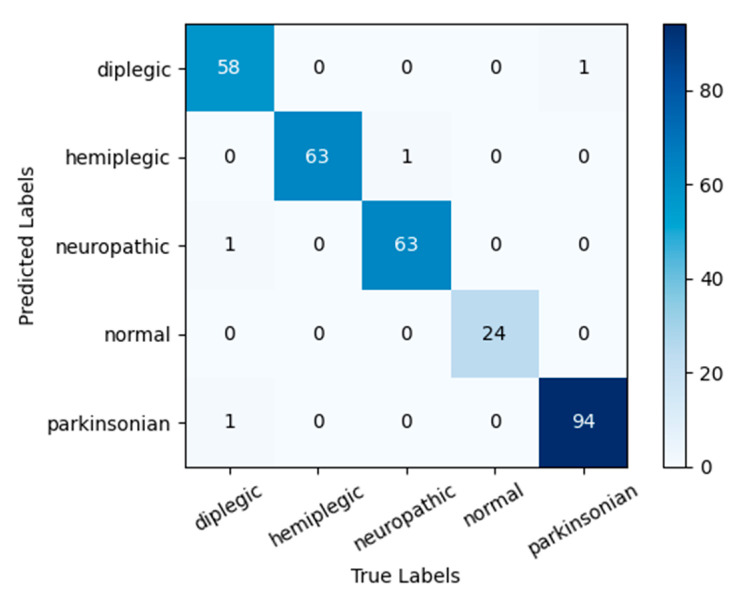
Confusion matrix of the model on the GAIT-IST dataset.

The confusion matrix obtained by applying the model NSVGT-ICBAM-FACN to the GAIT-IST dataset in this paper is shown in Figure 10. Two groups of diplegic gaits were incorrectly identified as neuropathic and Parkinsonian gaits, and one group of Parkinsonian gait was incorrectly identified as diplegic gait. This may be because the lower limbs move similarly in all three gaits, and the upper part of the body changes so little that the arm is not fully characterized in the side view. Thus, we found that of the five gaits, the three gaits mentioned above are not easily recognized.

Table 8 gives the test results of the NSVGT-ICBAM-FACN model on the GAIT-IST dataset. From the table, it can be seen that the precision of all five gaits is more than 98.31%, the sensitivity of diplegic gait is 100%, and the precision, sensitivity, and specificity of normal gait are all close to 100%. The results show that although abnormal gaits are not easy to recognize, the model can still recognize well the five gaits in the GAIT-IST dataset.

## 5. Conclusions

In this study, we introduce a novel abnormal gait recognition approach, NSVGT-ICBAM-FACN, aimed at achieving high accuracy while maintaining a lightweight model. We innovate by creating a new gait template, the FEI, which consolidates more discriminative information through the fusion of the GEI, GEnI, and the AEI using optimized weights determined via BGD. Key features are extracted using the ICBAM and integrated into a two-branch structure to generate effective fusion features. Moreover, DSC is employed to retain spatial information, allowing the network to adapt weights across various points. Ultimately, distinct pathological gaits are identified through a newly designed classification layer. Our algorithm synergistically incorporates feature fusion and loss, enhancing the model’s performance significantly. The proposed methodology demonstrates superior performance on both the self-constructed gait dataset and the GAIT-IST dataset, surpassing the majority of state-of-the-art methods in classification outcomes. Future endeavors will involve gathering abnormal gait data from multiple views to expand the dataset and further validate the efficacy of the developed gait template. Additionally, we aim to continue refining the model for increased lightweightness without compromising recognition accuracy. This algorithm not only aids doctors in diagnosing various pathologically induced gaits, but also serves as a benchmark for other abnormal gait recognition systems.

## Figures and Tables

**Table 1:** Gait characteristics of different gaits.

Gait Type	Gait Characteristics
Festinating gait	The subject’s body leaned forward with increasing speed and smaller steps [35].
Scissor gait	The subject’s legs flexed slightly at the hips and knees, with the knees and thighs hitting or crossing in a scissors-like movement [36].
Hemiparetic gait	The subject’s legs were swung outward in a semicircle [36].
Shuffling gait	Subjects dragged and took small steps [37].
Normal gait	Subjects walked normally without abnormal movements.

**Table 2:** Ablation experimental design.

Number	Single Branch	Dual Branch	ICBAM	DSC	Customized Head (Except DSC)
1	√	×	×	×	×
2	√	×	√	×	×
3	×	√	√	×	×
4	×	√	√	√	×
5	×	√	√	√	√

**Table 3:** Comparison of ablation experiment results.

Number	Acc (%)	Prec (%)	Sens (%)	Spec (%)	MF1
1	90.59	90.55	89.63	97.64	89.84
2	93.33	93.08	92.78	98.34	92.82
3	95.29	95.06	94.96	98.83	95
4	96.86	96.60	96.85	99.23	96.70
**5**	**98.43**	**98.18**	**98.38**	**99.62**	**98.26**

**Table 4:** Comparison of experimental results of different attention modules.

Type	Datasets	Parameters (Million)	Acc (%)
ECA	Our Dataset	2.8	96.86
SE	Our Dataset	3.01	97.25
CBAM	Our Dataset	3.01	97.65
**Proposed**	**Our Dataset**	**3.01**	**98.43**
ECA	GAIT-IST	2.68	98.04
SE	GAIT-IST	2.89	98.37
CBAM	GAIT-IST	2.89	98.37
**Proposed**	**GAIT-IST**	**2.89**	**98.69**

**Table 5:** Comparative experiments with different components in the new gait template.

Type	Acc (%)	Prec (%)	Sens (%)	Spec (%)	MF1
AEI	90.59	90.71	89.98	97.67	90.11
GEnI	96.08	95.80	95.60	99.03	95.67
GEI	95.69	95.59	94.77	98.93	95
**FEI**	**9** **8.43**	**98.18**	**9** **8.38**	**99.62**	**9** **8.26**

**Table 6:** Comparison of our model with the state-of-the-art models.

Method	Dataset	Data Type	Parameters (Million)	Flops (Billion)	Memory Usage (MB)	FPS (f·s^−1^)	Acc (%)
GhostNet [15]	GAIT-IST	SEI	2.6	0.176	-	-	98.10
VGG-19 [28]	GAIT-IST	SEI	139	19.6	532	76.38	97.40
Proposed	GAIT-IST	SEI	2.89	0.322	11.2	111.25	98.04
**Proposed**	**GAIT-IST**	**FEI**	**2.89**	**0.322**	**11.2**	**112.23**	**98.69**
VGG-19 [28]	Our Dataset	FEI	139	19.6	652	67.05	97.66
**Proposed**	**Our Dataset**	**FEI**	**3.01**	**0.42**	**11.7**	**111**	**98.43**

**Table 7:** Experimental results of the model on the self-constructed dataset.

Gait Type	Prec (%)	Sens (%)	Spec (%)
Festinating Gait	98.11	100	99.51
Scissor Gait	94.87	97.37	99.08
Normal Gait	100	98.53	100
Hemiparetic Gait	97.92	100	99.52
Shuffling gait	100	96	100

**Table 8:** Experimental results of the model on the GAIT-IST dataset.

Gait Type	Prec (%)	Sens (%)	Spec (%)
Diplegic Gait	98.31	96.67	99.59
Hemiplegic Gait	98.44	100	99.59
Neuropathic Gait	98.44	98.44	99.59
Normal Gait	100	100	100
Parkinsonian Gait	98.95	98.95	99.53

## Data Availability

The data presented in this study are available on request from the authors. The data are not publicly available due to the privacy concerns of subjects participating in the experiment.
